# Plasma levels of neurology-related proteins are associated with cognitive performance in an older population with overweight/obesity and metabolic syndrome

**DOI:** 10.1007/s11357-023-00764-y

**Published:** 2023-03-25

**Authors:** Martí Llaurador-Coll, Santiago Rios, Jesus F. García-Gavilán, Nancy Babio, Elisabet Vilella, Jordi Salas-Salvadó

**Affiliations:** 1 Universitat Rovira i Virgili, Departament de Medicina i Cirurgia, Reus, Spain; 2 Hospital Universitari Institut Pere Mata, Reus, Spain; 3 Institut d’Investigació Sanitària Pere Virgili-CERCA, Reus, Spain; 4 Universitat Rovira i Virgili, Departament de Bioquímica i Biotecnologia, Alimentació, Nutrició, Desenvolupament i Salut Mental ANUT-DSM, Reus, Spain; 5 Centro de Investigación Biomédica en Red de Fisiopatología de la Obesidad y Nutrición, Instituto de Salud Carlos III, Madrid, Spain; 6 Centro de Investigación Biomédica en Red de Salud Mental, Instituto de Salud Carlos III, Madrid, Spain

**Keywords:** Body mass index, Cognitive impairment, Metabolic syndrome, Older subjects, PREDIMED-Plus trial, Protein extension assay

## Abstract

Cognitive impairment is present in a broad spectrum of medical conditions and in aging. Here, we aimed to identify plasma proteins related to cognitive function in a sample of older adults with overweight/obesity and metabolic syndrome. A total of 129 subjects (mean age 64.7 years; 36% females) were grouped according to low (l-GCF, *N*=65) or high (h-GCF, *N*=64) global cognitive function and matched according to education, sex, age, and body mass index. Cognitive performance was assessed using neuropsychological tests. Plasma levels of 92 neurology-related proteins were assessed using a proximity extension assay. An elastic net regression analysis was used to identify proteins more associated with cognitive performance. Additionally, the protein expression levels were compared between the two groups by means of a *t*-test with false discovery rate correction. Pearson correlations were used to assess associations between the protein levels and scores from the neurocognitive tests. Six proteins (alpha-2-MRAP, HAGH, Siglec-9, MDGA1, IL12, and EDA2R) were identified as potential contributors to cognitive performance, remaining significantly increased in l-GCF compared to h-GCF participants after correction for multiple testing. Negative correlations (*r*= −0.23 to −0.18, i.e., lower protein levels, higher cognitive function) were found between global cognitive function and Siglec-9, NMNAT1, HAGH, LXN, gal-8, alpha-2-MRAP, IL12, PDGF-R-alpha, NAAA, EDA2R, CLEC1B, and LAT. Mini-mental state examination *z* scores showed the strongest correlations with protein levels, specifically negative correlations with CLEC1b, LXN, LAT, PLXNB3, NMNAT1, gal-8, HAGH, NAAA, CTSS, EZR, KYNU, MANF (*r*=−0.38 to −0.26) and a positive correlation with ADAM23 (*r*= 0.26). In summary, we identified several plasma proteins that were significantly associated with cognitive performance in older adults with obesity and metabolic syndrome, although further research is needed to replicate the results in larger samples and to include a predictive perspective.

**Supplementary Information:**

The online version contains supplementary material available at 10.1007/s11357-023-00764-y.

## Introduction

Cognitive processes, which encompass language, imagination, perception, and planning, can affect every aspect of life, from school to work and relationships. Classically, cognitive performance is assessed by means of neuropsychological tests, which, although widely used in clinical settings, require considerable time, expertise, and caution when interpreting and comparing results across cultures. Aside from aging-associated cognitive decline, there is a wide range of medical conditions that can be associated with cognitive impairment (CI). In neurodegenerative diseases, CI is irreversible and often precedes dementia [1]. Medical conditions linked to CI include psychiatric disorders (for instance, schizophrenia and bipolar disorder) [2] as well as diabetes [3], obesity [4], and other inflammatory diseases [5]. CI is still an important obstacle to the treatment of these medical conditions, significantly influencing the overall outcome and functional recovery, and is strongly related to the patients’ quality of life [6]. Cognitive performance has an important genetic basis [7] and largely depends on age [8], in addition, traumatic experiences throughout the lifespan can have a negative impact [9]. Conversely, some modifiable environmental factors, such as education and lifestyle, may also play an important role.

Conventional clinical evaluation tools for CI are limited in applicability, while easily accessible biomarkers for CI are lacking. Therefore, identifying CI protein biomarkers could be useful in predictive medicine and in monitoring cognitive performance in clinical interventions. Although cerebrospinal fluid (CSF) is the primary fluid of choice for brain dysfunction biomarker discovery, the lumbar puncture procedure to obtain CSF is not routinely performed. Instead, blood has become a common biofluid for brain biomarker discovery [10]. Highly sensitive and throughput molecular methods allow the simultaneous quantification of hundreds of proteins in plasma and the selection of tissue-specific panels of novel biomarkers. The proximity extension assay (PEA) is a novel method that combines immunological detection with quantitative polymerase chain reaction (qPCR) [11]. Through this combination, substantial scalability, sensitivity, and specificity can be achieved, where relative quantification of multiple proteins present in a wide concentration range is possible. PEA neurology-based panels have been used to identify protein biomarkers of neurodegeneration and cognitive decline. Recent studies using PEA on plasma or CSF have identified proteins altered in cerebral hereditary adrenoleukodystrophy [12], Alzheimer’s disease (AD) [13, 14], parkinsonian syndromes [15] and, more specifically, mild cognitive impairment (MCI) in posttraumatic stress disorder [16] and HIV [17] patients. To the best of our knowledge, only one study used a PEA neurology-based panel to specifically discover protein biomarkers of age-related cognitive impairment in two large cohorts [18]. The study reported that plasma levels of 22 out of the 92 neurology-related proteins are associated with cognitive ability in older age and that in some cases, the associations were mediated by brain gray matter volume.

Data from trials testing different diet and exercise interventions for both obesity and diabetes [4, 19, 20] have demonstrated that cognitive performance can be partially recovered and cognitive decline delayed. One such trial is the PREDIMED-Plus study, where physical exercise [21], adherence to the Mediterranean diet (MedDiet) [20], and glycemic regulation [3] were associated with better cognitive function. PREDIMED-Plus is a primary prevention cardiovascular study testing the effect of a lifestyle intervention program with an energy-restricted diet, weight-loss goals, and physical activity promotion in elderly participants with overweight/obesity and metabolic syndrome (MetS) [22]. In the present study, a subsample of the PREDIMED-Plus trial was used to explore and identify plasma proteins associated with CI at baseline. First, in a cross-sectional design based on cognitive performance, we compared the protein expression levels in plasma between low and high performers, and second, using the whole sample, we assessed the correlation between plasma protein expression levels and scores from the cognitive tests.

## Materials and methods

### Study design and participant selection

We designed a cross-sectional study comparing two groups of PREDIMED-Plus participants at baseline according to their global cognitive function (GCF) score determined as a composite score of the evaluated cognitive tests. The PREDIMED-Plus (PREvención con DIeta MEDiterránea) trial is an ongoing, multicenter, parallel, randomized controlled clinical trial conducted in Spain to explore the primary prevention of cardiovascular disease. Participants were community-dwelling adults (55–75 years) with overweight/obesity (body mass index (BMI) 27 ≥ and <40 kg/m^2^) who met at least three criteria of MetS [23]. The study protocol has been described and published elsewhere [22] and can be accessed at http://www.predimedplus.com. All participants provided written informed consent, and the study protocol and procedures were approved by the ethics committee (CEIC Hospital Universitari de Sant Joan). The trial was registered in 2014 at the International Standard Randomized Controlled Trial (http://www.isrctn.com/ISRCTN89898870).

**Fig. 1:**
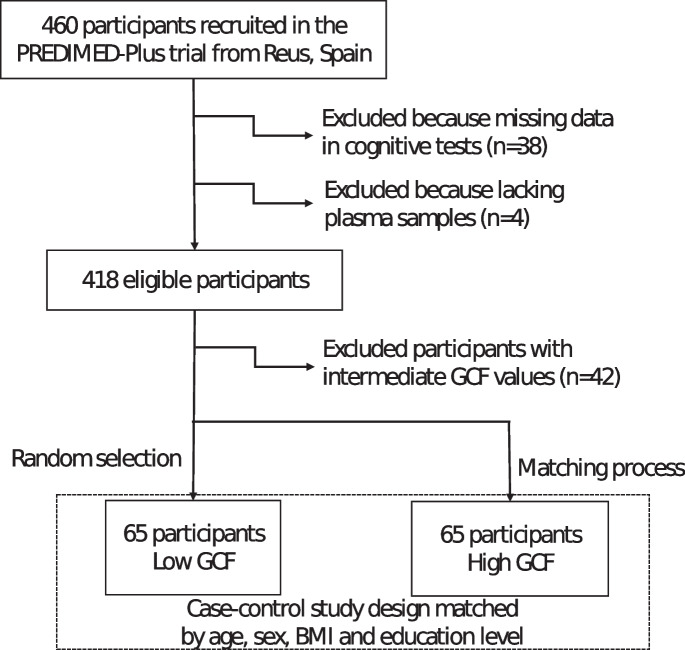
Flow diagram of the study design and participant selection*. Created with Microsoft PowerPoint.*

For the present study, only those participants belonging to the Reus recruiting center (Spain) that participated in the baseline assessment (previous to the trial intervention) and accomplished eligibility criteria were selected (*n*=418). Participants were categorized according to the average z score of the global cognitive function (zCGF) value between baseline and the 2-year follow-up. zGCF was obtained by the addition of the eight previously standardized cognitive test scores. Half of the participants with a lower zCGF were classified into the “l-GCF” group, and the other half with a higher zGCF were classified into the “h-GCF” group. To increase the differences between both groups, 42 participants with intermediate values (percentile between 45 and 55) were excluded. A total of 65 of the 189 participants in the l-GCF group were randomly selected and then matched 1:1 to participants in the h-GCF group based on age (< 65 years and ≥ 65 years), sex (female or male), BMI (< 32.58 and ≥ 32.58 kg/m^2^), and education level (primary school, high school, college or technician). Figure 1 shows the participant selection flow chart.

### Descriptive data and sample collection

At baseline, participants provided general descriptive information by answering general questionnaires, in a face-to-face interview, on sociodemographic variables (sex, age, level of education, and civil status), lifestyle (smoking habits, physical activity), disease history, and drug use, among others. Leisure-time physical activity was evaluated with the validated REGICOR questionnaire [24]. Adherence to an energy-reduced Mediterranean diet was assessed by a validated 17-point diet score [25]. Additionally, anthropometric variables (weight, height, waist circumference) and blood samples were obtained in fasting conditions by trained nurses.

### Cognitive assessment

Eight cognitive tests were administered by trained personnel at baseline and after two years of follow-up. Baseline measurements were used to analyze the associations between cognition and measured proteins. The mini-mental state examination (MMSE) and the clock drawing test (CDT) were used to assess global cognition, and the digit span test forward (DST-f) and backward (DST-b) section, the Verbal Fluency Test animals (VFT-a) and “p” (VFT-p) version, and the trail making test part A (TMT-A) and part B (TMT-B) were used to explore more specific cognitive domains, all of which are described in Supplementary Material 1. The final score of each cognitive test was standardized using the mean and the standard deviation of the population scores. The *z* scores for TMT-A and TMT-B were inverted so that lower scores were indicators of poorer performance, as is the case for the rest of the tests. For the eight cognitive tests, a GCF *z* score (using the mean of the standardized scores of the eight tests) [26] and three cognitive function domain *z* scores were derived: (a) general cognitive function (zGenCF) domain using the MMSE and CDT tests; (b) executive function (zExF) domain using the VFT-a, VFT-p, TMT-B, and DST-b tests; and (c) attention (zAtt) domain using the TMT-A and DST-f tests [27, 28].

### Biochemical measurements

Blood was collected in serum and EDTA-containing tubes, and after centrifugation, serum and plasma aliquots were stored at −80°C in the “Institut d’Investigació Sanitària Pere Virgili” (IISPV) biobank until needed for biochemistry analysis. Serum glucose, triglycerides, total cholesterol, and HDL-cholesterol levels were measured using standard enzymatic methods, and LDL-cholesterol concentrations were calculated with the Friedewald formula [29] and used for the MetS criteria.

Coded 50 μl plasma samples were sent randomly distributed in 96-well plates to Olink Proteomics Uppsala headquarters and were assessed in one batch. A total of 92 neurology-related protein biomarkers were measured by PEA technology (Olink® Proteomics, Uppsala, Sweden). Olink internal controls are spiked into the assay to monitor every step of the process, while external controls are run on each plate to monitor variation between plates. The sensitivity is similar to that of ELISA or better (pg/mL), and the average intra-assay %CV, a measure of precision, is <10%. The data were preprocessed by Olink® using NPX Manager software [11]. Protein levels are presented as normalized protein expression (NPX) units. NPX is Olink Proteomics’ arbitrary unit on the log2 scale of the quantification cycle (Cq) in the sample, where a larger number represents a higher protein level. Linearization of data was performed by the 2^NPX^ formula with a background level close to zero [11]. The measured proteins were selected based on previous studies [18, 30]. The quality control of each sample was assessed by evaluating the deviation from the median value of the controls and the detection range for each protein. One sample belonging to the h-GCF group was excluded because it did not pass the PEA assay quality control. Measured proteins (MAPT, LXN, and β-NGF) with a high percentage (>98%) of data values below the limit of detection (LOD) are reported.

### Statistical analysis

Descriptive data analysis was performed using counts and percentages for categorical variables, mean and standard deviation (SD) values for quantitative traits, and median and interquartile range (IQR) values for nonparametric quantitative traits. Outcomes were compared against l- and h-GCF groups by chi-square, Student’s *t*-test, or the Mann–Whitney *U* test. NPX levels are described as the mean and SD values, and the l-GCF and h-GCF groups were compared by Student’s *t*-test adjusted using the FDR method. Furthermore, the correlations of NPX values for every protein level with the baseline *z* score of every cognitive test and of the three cognitive function domains were calculated using Pearson coefficients, and data are represented as correlation heatmaps with a hierarchical clustering dendrogram (“ComplexHeatmap” R package).

Because of the highly collinear nature of data collected with the same method, logistic (binomial) regression models were used with the elastic net penalty (“glmnet” R package) to select the proteins more associated with each GCF category. A 10-fold cross-validation (CV) approach was performed by splitting the data into training and validation sets containing 80% and 20% of the sample, respectively, to identify the optimal value of the tuning parameter. A 10-fold CV was performed within the training sets to determine the optimal value of the tuning parameter (*λ*) to yield the minimum misclassification error using the argument *s* = “lambda.min” in the cv.glment function. Additionally, the *α* parameter was evaluated from 0 (i.e., a ridge regression) to 1 (i.e., a LASSO regression) in 0.1 increments to test the best parameter for these analyses, with this value equal to 0.8. For reproducibility purposes, protein coefficients were estimated using 10 iterations of the 10-CV elastic net regression with all participants. The coefficient values are the mean of the proteins selected in each iteration.

All statistical procedures were carried out with Stata 14.2 software for Windows (Stata Corp.) and R software v4.1.0 (www.R-project.org) (R Core Team, 2021).

## Results

### Sample description

The general characteristics of participants in both the l- and H-GCF groups are shown in Table 1. According to the screening tests, 5 participants from the l-GCF group showed signs of MCI (MMSE score between 20 and 24). The remaining participants in both groups were in the normal range. The zGCF was -0.49 ± 0.50 and 0.52 ± 0.28 in the l- and h-GCF groups, respectively. Participants in the l-GCF category showed lower values in all cognitive function domains and each cognitive test (*p* value <0.001) than those in the h-GCF category. There were no more differences between participants in both categories. In general, participants were mainly males (64%), had an average age of 64.7 years, had an average BMI of 32.8 kg/m^2^, were married, nonsmokers, and had a low education level. The prevalence of the following MetS criteria was observed in the total selected population: 91% of participants had an increased waist circumference (> 102 cm in men and > 88 cm in women), 77% had hypertriglyceridemia (serum triglycerides ≥ 150 mg/dL or drug treatment for elevated triglycerides), 62% had low HDL-cholesterol levels (≤ 40 mg/dL in men and ≤ 50 mg/dL in women or drug use for low HDL), 95% had hypertension (systolic blood pressure ≥ 130 mmHg), and 57% had hyperglycemia (fasting plasma glucose level ≥ 100 mg/dL or hypoglycemic treatment).

**Table 1:** Baseline characteristics the study participants in different cognitive function categories

	l-GCF (*n*=65)	h-GCF (*n*=64)	*p* value*
zGCF	−0.49 ± 0.50	0.52 ± 0.28	<0.001
Cognitive domains *z* scores			
zgenCF	−0.50 ± 0.99	0.34 ± 0.42	<0.001
zExF	−0.48 ± 0.57	0.62 ± 0.44	<0.001
zAtt	−0.51 ± 0.75	0.53 ± 0.48	<0.001
Cognitive test scores			
MMSE	28 (27–29)	29 (29–30)	<0.001
CDT	6 (4–6)	6 (6–7)	<0.001
VFT-a	13 (11–16)	17 (15–19)	<0.001
VFT-p	10 (8–12)	14 (13–17)	<0.001
TMT-A^a^	65 (53–90)	38 (30–46)	<0.001
TMT-B^a^	164 (126–236)	88 (69–104)	<0.001
DST-f	8 (7–9)	10 (9–12)	<0.001
DST-b	4 (3–5)	7 (6–8)	<0.001
Age (years)	65.38 ± 5.17	63.84 ± 5.47	0.103
Sex, % women	38.46 (25)	32.81 (21)	0.503
BMI (kg/m^2^)	33.16 ± 3.05	32.14 ± 3.45	0.075
Diabetes, %	21.54 (14)	12.50 (8)	0.172
Waist circumference (cm)	108.86 ± 11.25	107.32 ± 10.23	0.504
Physical activity (MET min/week)	2097.90 (1324.01–3314.69)	2083.92 (867.13–3370.63)	0.708
17-point MedDiet score	7.91 ± 2.02	7.59 ± 2.49	0.433
Level of education, *N* (%)			0.993
High education	10.77 (7)	10.94 (7)	
Medium education	36.92 (24)	35.94 (23)	
Low education	52.31 (34)	53.13 (34)	
Civil status, *N* (%)			0.107
Single, divorced or separated	18.46 (12)	6.25 (4)	
Married	73.85 (48)	85.94 (55)	
Widower	7.69 (5)	7.81 (5)	
Smoking habits, *N* (%)			0.962
Smoker	16.92 (11)	15.63 (10)	
Former smoker	44.62 (29)	46.88 (30)	
Never smoker	38.46 (25)	37.50 (24)	
Serum glucose (mg/dL)	106.57 ± 19.63	104.28 ± 19.35	0.506
Triglycerides (mg/dL)	185.03 ± 101.37	183.33 ± 98.36	0.923
LDL cholesterol (mg/dL)	117.55 ± 29.44	127.84 ± 36.70	0.101
HDL cholesterol (mg/dL)	47.63 ± 12.14	49.23 ± 10.04	0.415

Descriptive data are expressed as a percentage (counts) for categorical variables, mean ± SD for quantitative variables, and median (Q1-Q3) for quantitative nonparametric variables

*p value was calculated using Chi-square, Student’s *t*-test, or Mann–Whitney *U* tests as appropriate

*l-GCF* low global cognitive function, *h-GCF* high global cognitive function, *zGCF z* score of global cogitive function, *zgenCF z* score of general cognitive function, *zExF z* score of executive function, *zAtt z* score of attention function, *BMI* body mass index, *MedDiet* Mediterranean diet, *HbA*_*1c*_ glycated hemoglobin, *MMSE* mini-mental state examination, *CDT* clock drawing test, *VFT-a* verbal fluency test animal category, *VFT-p* verbal fluency test letter “p”, *TMT-A* trail making test part A, *TMT-B* trail making test part B, *DST-f* digit span test forward section, *DST-b* digit span backward section, *MET* metabolic equivalent task, *LDL* low-density lipoprotein, *HDL* high-density lipoprotein

^a^TMT-A and TMT-B test scores are inverse to neuropsychological assessment: the greater the score, the lower the cognitive performance

The range of protein levels in the whole sample (Supplementary Figure S1) was from 0.308 NPX (for Beta-NGF) to 10.894 NPX (for RGMA). The mean ± SD for protein levels overall is 5.81 ± 2.18. Furthermore, for each protein, we compared the number of participants with an expression value below the first decile between l- and h-GCF groups (Supplementary Table S3), and only IL12 showed a nominal *p*<0.05.

### Comparison of protein levels between the l- and h-GCF groups

After the *t*-test, seven proteins showed significantly higher levels in the I-GCF group compared to the h-IGF group (Supplementary Table S1): alpha-2-MRAP, MDGA1, Siglec-9, HAGH, EDA2R, IL12, and MSR1. After FDR adjustment these differences become non-significant.

**Fig. 2:**
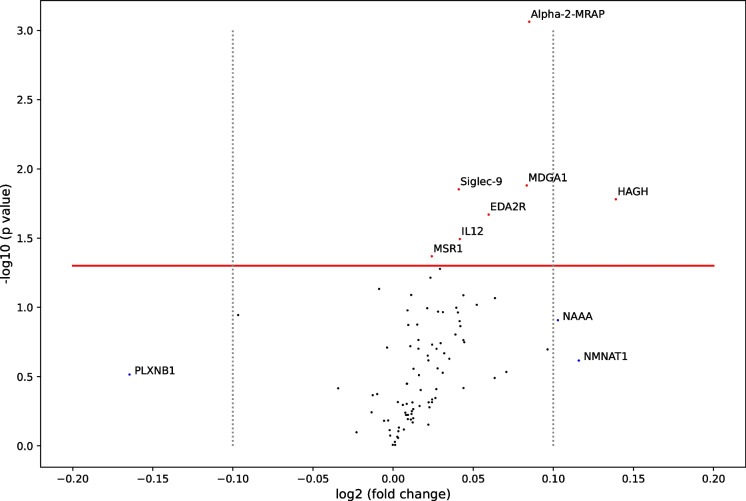
Volcano plot of normalized protein expression levels (NPX) between the l-GCF and h-GCF groups. Volcano plot shows log_2_ fold-change (FC) values of l-GCF with respect to h-GCF plotted against log_10_ *p* values between the two groups as calculated with Student’s *t*-test. Red dots represent proteins with significant differential expression between the two groups. A red line is plotted at -log_10_ *p* value = 1.3 (corresponding to a *p* value of 0.05), and two gray-dotted lines are plotted at log_2_ FC values of −0.1 and 0.1 (corresponding to a difference of ±7.17% between groups)*. Created with “Matplotlib” Python library*

Figure 2 shows the log2 fold-changes and log10 *t*-test *p* values between GCF groups. NAAA, NMNA1, and HAGH were positively associated with l-GCF and showed log2 FC >0.1, but only HAGH was statistically significant between GCF groups. In addition, only PLXNB1 showed a log2 fold-change (FC) value lower than −0.1, but the difference was not statistically significant.

Table 2 shows the statistical comparison between the study groups of the 9 proteins that were selected by binomial elastic net regression. Of these, six proteins were associated with the l-GCF category: alpha-2-MRAP (coef: −0.42), HAGH (coef: −0.39), Siglec-9 (coef: −0.27), MDGA1 (coef: −0.18), IL12 (coef: −0.17), and EDA2R (coef: −0.14), and the mean value of NPX between the two groups was significantly different after adjusting the *p* value using FDR. Conversely, three proteins were more associated, although without reaching statistical significance, with the h-GCF category: NEP (coef: 0.44), NBL1 (coef: 0.27), and RGMA (coef: 0.17).

**Table 2:** Normalized expression levels of the 9 selected proteins based on elastic net regression for all participants and between global cognitive function categories

Protein name	Symbol	UniProt ID	l-GCF	h-GCF	∆ (l-h)	FDR*
Alpha-2-macroglobulin receptor-associated protein	Alpha-2-MRAP	P30533	8.84 (8.58, 9.11)	8.34 (8.20, 8.47)	0.50	0.008
Hydroxyacylglutathione hydrolase, mitochondrial	HAGH	Q16775	3.98 (3.74, 4.22)	3.61 (3.43, 3.79)	0.37	0.037
Sialic acid-binding Ig-like lectin 9	Siglec-9	Q9Y336	5.02 (4.94, 5.10)	4.88 (4.80, 4.96)	0.14	0.037
MAM domain-containing glycosylphosphatidylinositol anchor protein 1	MDGA1	Q8NFP4	5.75 (5.57, 5.92)	5.42 (5.24, 5.61)	0.33	0.037
Tumor necrosis factor receptor superfamily member 27	EDA2R	Q9HAV5	4.85 (4.71, 4.98)	4.65 (4.55, 4.75)	0.20	0.038
Interleukin-12	IL12	P29460, P29459	8.98 (8.83, 9.13)	8.72 (8.54, 8.90)	0.26	0.048
Neuroblastoma suppressor of tumorigenicity 1	NBL1	P41271	5.43 (5.40, 5.46)	5.47 (5.44, 5.49)	−0.04	0.095
Neprilysin	NEP	P08473	3.17 (2.97, 3.37)	3.39 (3.20, 3.58)	−0.22	0.128
Repulsive guidance molecule A	RGMA	Q96B86	10.89 (10.80, 10.97)	10.90 (10.83, 10.98)	−0.01	0.770

Descriptive data are expressed as the mean (confidence interval)

*p value was calculated using Student’s *t*-test adjusted by the false discovery rate (FDR) method

### Association between protein levels and scores on the cognitive tests

**Fig. 3:**
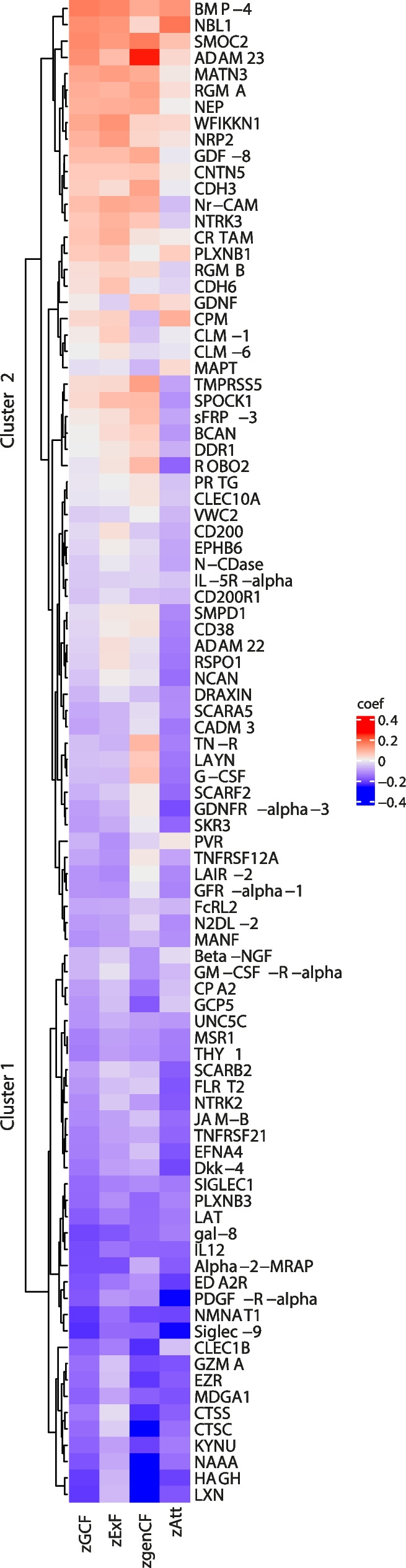
Heatmap of the Pearson correlation coefficient values between normalized protein expression levels (NPX) and *z* scores of the global cognitive function (zGCF), executive function (zExF), general cognitive function (zGenCF), and attention (zAtt) domains. Pearson correlation coefficient values are colored with a blue-red scale. Proteins are ordered by the hierarchical clustering method*. Created with “ComplexHeatmap” R package*

**Fig. 4:**
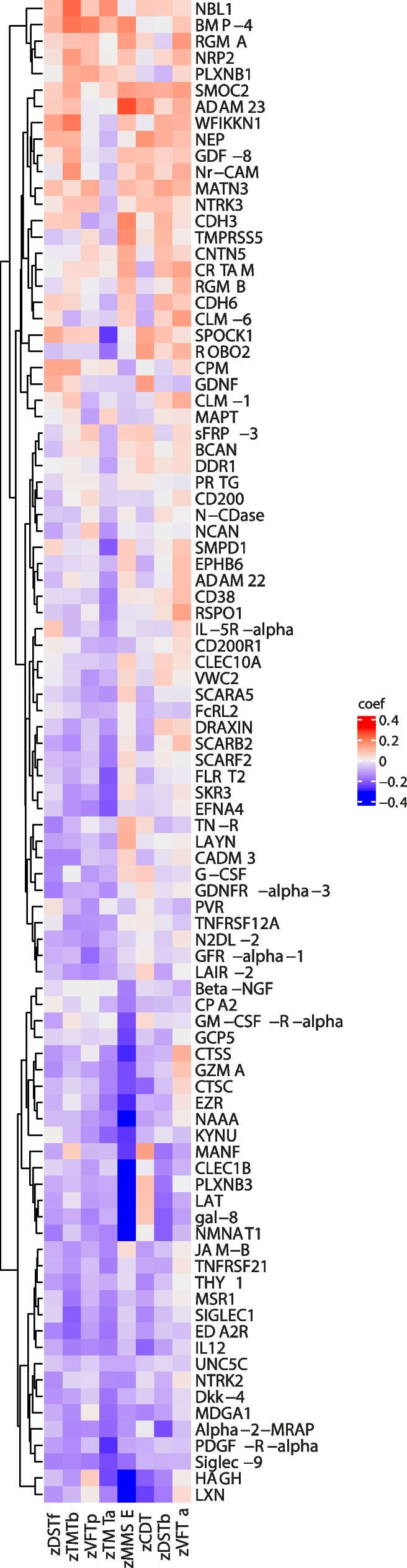
Heatmap of the Pearson correlation coefficient values between normalized protein expression levels (NPX) and the standardized values of the eight cognitive tests. Pearson correlation coefficient values are colored with a blue-red scale. Proteins are ordered by the hierarchical clustering method*. Created with “ComplexHeatmap” R package*

We explored the relationship between protein levels and cognitive performance by conducting correlation analysis first using the global and cognitive domain *z* scores (Fig. 3) and then each test *z* score separately (Fig. 4). The corresponding Pearson correlation coefficients are shown in Supplementary Table S2. The heatmap with the clustering analysis in Fig. 3 shows that cluster 1 was composed of the strongest negative associations between NPX and *z* scores, and cluster 2 was composed of a mixture of positive and mild negative associations. The strongest correlations (*r* > │0.25│) were observed between zGenCF and LXN (*r*= −0.37), HAGH (*r*= −0.33), NAAA (*r*= −0.27), CTSC (*r*= −0.26), and ADAM23 (*r*= 0.26) and between zAtt and Siglec-9 (*r*= −0.26) and PDGF-R-alpha (*r*= *-*0.26). Regarding zGCF only statistically significant negative correlations (*r*= −0.23 to *r*=−0.18) were found with Siglec-9, NMNAT1, HAGH, LXN, gal-8, alpha-2-MRAP, IL12, PDGF-R-alpha, NAAA, EDA2R, CLEC1B, and LAT (Supplementary Table S2 and Fig. 3). Regarding the tests assessed individually (Fig. 4), the strongest associations (*r* >│0.25│) were found with zMMSE and CLEC1b (*r*= −0.38), LXN (*r*= −0.36), LAT (*r*= −0.36), PLXNB3 (*r*= −0.36), NMNAT1 (*r*= −0.35), gal-8 (*r*= −0.35), HAGH (*r*= −0.33), NAAA (*r*= −0.33), CTSS (*r*= −0.29), EZR (*r*= −0.28), KYNU (*r*= −0.27), MANF (*r*= −0.26), and ADAM23 (*r*= 0.26); between the zCDT and LXN (*r*= −0.25); and between the zTMT-A and PDGF-R-alpha (r= −0.27) and SPOCK1 (*r*= −0.26). Negative correlations mean that lower levels of these proteins were associated with a higher cognitive performance. Notably, the strongest positive correlation, that is higher protein levels are found with higher cognitive function (MMSE), was observed for ADAM23.

## Discussion

Here, we showed a significant increase in NPX in 6 proteins (alpha-2-MRAP, HAGH, Siglec-9, MDGA1, EDA2R, and IL12) in the l-GCF group of older adults with overweight/obesity and MetS from the PREDIMED-Plus cohort. Moreover, through correlation analysis with relevant coefficient values (*R* >│0.25│), we show significant inverse relationships (lower protein level, higher cognitive function) between NPX and general cognitive domain (12 proteins, Siglec-9, NMNAT1, HAGH, LXN, gal-8, alpha-2-MRAP, IL12, PDGF-R-alpha, NAAA, EDA2R, CLEC1B, and LAT) and attention (6 proteins LXN, HAGH, NAAA, CTSC, Siglec-9, and PDGF-R-alpha). The individual test that showed stronger correlations was the MMSE, with inverse relationships with 12 proteins (CLEC1b, LXN, PLXNB3, NMNAT1, gal-8, HAGH, NAAA, CTSC, EZR, KYNU, and MANF) and a direct relationship with one (ADAM23). As expected, the five proteins associated with the l-GCF category were inversely correlated to zGCF score. The protein with the highest differential expression between both GCF groups was alpha-2-MRAP. Alpha-2-MRAP, also known as receptor-associated protein (RAP), is a chaperone and ligand-binding inhibitor of the LDL receptor family of proteins [31]. Among the lipoprotein receptors, one main target for RAP is LDL receptor-related protein 1 (LRP1), which is involved in several processes, such as clearance of alpha-2-MRAP and lipoproteins from plasma and clearance of amyloid β peptides and cholesterol transport in the brain. Thus, RAP, through its interaction with LRP1, is a key protein in brain cholesterol homeostasis [32] and in the pathophysiology of amyloid β deposition in Alzheimer’s disease (AD) [31]. A recent study using a mice diabetes model found that hyperglycemia can impair Aβ efflux in the brain microvasculature by downregulating the expression of LRP1 and resulting in cognitive impairment [33]. Since participants’ metabolic and biometric parameters were similar between the two GCF study groups, we can postulate that the differences observed in the protein expression in plasma are not due to peripheral metabolic changes but rather are related to brain processes. Regarding this, a previous work using PEA technology to quantify proteins in plasma-isolated exosomes found alpha-2-MRAP at a higher concentration in neuron-derived exosomes (NDEs) than in other exosomes present in plasma [34]. Moreover, studying brain samples from patients with AD and healthy controls, an inverse relationship between RAP and amyloid β was found [35], but in young adults with genetic risk for AD, RAP in brain tissue was found to be increased [36]. These results indicate that RAP may be related to neurodegenerative processes, although more investigation is needed in the future. Alpha-2-MRAP also modulates the binding of alpha-2-macroglobulin (alpha-2-M) to LRP1 [37]. Interestingly, alpha-2-M, a plasma proteinase inhibitor was found positively correlated with endothelial dysfunction in patients with a history of stroke [38].

Hydroxyacylglutathione hydrolase (HAGH) showed the highest fold-change, although not the most significant, between both study GCF groups, with increased expression in the l-GFC group. Using the same PEA Olink assay as in the present study, HAGH was found to be increased in plasma from individuals with HIV-associated cognitive impairment [17], in amyloid β-positive individuals with mild cognitive impairment (MCI) and AD [39], and in APO E4-carrier individuals with AD [40]. The HAGH protein, also known as glyoxalase-2, is an enzyme involved in the glyoxalase system that plays a key role in the control of oxidative stress. The glyoxalase system participates in the detoxification of glycolysis byproducts, particularly targeting the cytotoxic metabolite methylglyoxal. Methylglyoxal levels are elevated in plasma in the context of various disease conditions, including hyperglycemia, and form reactive oxygen species causing oxidative stress. Moreover, methylglyoxal is the precursor of glycation end-products, which are implicated in AD through regulation of amyloid precursor protein (APP) expression [40]. It has been hypothesized that increased plasma levels of HAGH in prodromal stages might be a general stress response [40] and, specifically in the brain, a mechanism to decrease methylglyoxal accumulation due to neurodegenerative processes. In addition, HAGH expression levels may reflect brain microvascular endothelial dysfunction related to hyperglycemia [41]. Since individuals in our study had no overt dementia and since HAGH levels have been found to be increased in NDEs [17], we can speculate that HAGH could be a prognostic biomarker for cognitive decline, although more studies are warranted to prospectively confirm these results in other cohorts.

Sialic acid–binding immunoglobulin-like lectin 9 (Siglec-9) is a transmembrane protein that binds sialic acid and favors cell–cell adhesion in immunological processes [42]. Our results of higher levels of Siglec-9 in the l-GCF group are in line with the results found in two cohorts of mainly healthy older individuals showing an inverse relationship between Siglec-9 and fluid cognitive ability [18]. Higher levels of Siglec-9 in plasma extracellular vesicles (EVs) were found in AD patients than in patients with MCI [14]. Siglec-9, which is expressed in the brain, was found to bind glioblastoma-derived EVs [43], and a potential role in neuroinflammation has been suggested [44]. Moreover, Siglec-9 expression increases with high glucose exposure of endothelial cell in vitroconcomitant with proatherogenic processes [45].

MAM domain–containing glycosylphosphatidylinositol anchor protein 1 (MDGA1) is a cell surface protein expressed predominantly in the brain that is involved in cell adhesion, migration, and axonal guidance during neurodevelopment and in the formation and maintenance of inhibitory synapses [16]. Our findings support results from a previous study that found that MDGA1 was upregulated in cognitively impaired subjects with and without posttraumatic stress disorder [16]. Interestingly, a recent study in mice showed that the overexpression of MDGA1 in hippocampal CA1 neurons impaired novel object-recognition memory through a complex interaction with amyloid precursor protein-mediated synaptic inhibition [46].

Ectodysplasin A2 receptor (EDA2R) is the transmembrane receptor for the ectodysplasin (EDA) A2 isoform, and the ligand-receptor complex participates in multiple signaling pathways [47]. EDA is mainly expressed in the liver and is considered a hepatocytokine that can be secreted into the circulatory system to participate in energy and glycolipid metabolism. Interestingly, the EDA-A2/EDA2R complex has been implicated in the regulation of glucose metabolism in individuals with diabetes mellitus, and serum EDA-A2 levels have been related to BMI and obesity [47]. We found significantly elevated levels of EDA2R in l-GCF compared to h-GCF, and similar results (higher levels of a protein associated with CI) were found in two previous studies [16, 18]. However, no study to date has suggested a specific role for the EDA-A2/EDA2R complex in brain function.

Interleukin-12 (IL12) is a cytokine expressed by activated macrophages that acts on T and natural killer cell activation and has been shown to induce long-term immune protection [48]. IL12 participates in cell adhesion, vascular remodeling, and repair processes [49] in the pigment epithelium–derived factor (PEDF) signaling system with antiangiogenic, antitumorigenic, and neurotrophic functions. We observed higher IL12 NPX in the l-GCF group. In line with our results, increased levels of IL12 were found to be associated with reduced performance in processing speed in elderly individuals [50] and with poorer neurocognitive performance as assessed by the MATRICS Consensus Cognitive Battery in patients with schizophrenia [51]. Nevertheless, IL12 was found to be associated with slower cognitive decline in patients with elevated amyloid β [52]. IL12 inhibition with a monoclonal antibody improved coronary, arterial, and myocardial functioning psoriasis patients [53]. Although it is largely accepted that IL12 is an important immunomodulator, its role in cognitive performance deserves further investigation.

In summary, inverse associations between the expression levels of alpha-2-MRAP, HAGH, EDA2R, Siglec-9, MDGA1, and IL12 and cognitive impairment have been demonstrated in different studies, including ours, and are suggestive of possible future use of these proteins as biomarkers for cognitive performance in older adults. Moreover, HAGH and Siglec-9 were among the proteins showing significant inverse correlations with global cognitive scores and MMSE scores. ADAM23 was the only protein found to be significantly directly correlated with zGenCF and MMSE scores. ADAM23 is a metalloprotease without protease activity that is predominantly expressed in the brain. Together with ADAM22, these proteins function as receptors for leucine-rich glioma-inactivated (LGI) protein family members, which are neuronal proteins secreted into the synaptic cleft. The interaction of LGIs with ADAM22 and ADAM23 is involved in key processes in the brain, such as myelination and synaptic transmission [54]. Moreover, ADAM 23, which negatively regulates potassium current was found upregulated and associated with the onset of hypertension in a hypertensive rat model study [55].

Our study has some limitations that need to be mentioned. First, the limited sample size, which did not preclude having enough power to detect differences between the two study groups, was not large enough to stratify according to sex for instance to further explore the relationship between proteins and cognition. Second, we did not adjust the NPX values for known expression quantitative trait loci (eQTLs) for the 92 proteins in the panel used [56], and we should mention that at least the expression of MDGA1 and Siglec-9 showed a dose-dependent effect of rare alleles at loci r6938061 and r4857414, respectively [56]. Third, the cognitive assessment was restricted to generic measures such as general cognitive function, executive function, and attention; however, working memory, reasoning, or social cognition, for instance, were not assessed. Fourth, the cross-sectional design of our study did not allow us to test prospective associations. Sixth, the expression values of three proteins (MAPT, beta-NGF, and LXN) were under the LOD for the PEA technique, and it is necessary to use caution in interpreting the results for these 3 proteins.

Conversely, we consider the experimental design and the sample selection as the main strengths of our study. This pilot study indicates that the plasma proteins alpha-2-MRAP, HAGH, Siglec-9, MDGA1, EDA2R, and IL12 are negatively associated with cognitive performance in a sample of older adults with overweight/obesity and MetS. However, the lack of generalizability of our findings to other populations makes it essential to reproduce and validate our results in other similar populations and to perform longitudinal prospective designs to investigate whether blood-based biomarkers can predict the short-term and/or long-term evolution of cognitive function.

### Supplementary information

ESM 1

ESM 2

ESM 3

ESM 4

ESM 5
