# Supplementary material for: Plasma levels of neurology-related proteins are associated with cognitive performance in an older population with overweight/obesity and metabolic syndrome
Source: GeroScience. 2023 Mar 25;45(4):2457–70. doi: 10.1007/s11357-023-00764-y (PMC10651568; doi:10.1007/s11357-023-00764-y)
Supplement: Supplementary file 1 — ESM 1 [file 11357_2023_764_MOESM1_ESM.pdf]

Supplementary Material 1 Additional cognitive assessment description

The MMSE was developed as a general cognitive test to screen for cognitive decline [1]; the validated Spanish version [2] was used in the present study. This test is composed of two parts. The first part requires only vocal responses and covers orientation, memory, and attention capacities. The second part requires the ability to name object, follow verbal and written commands, write a sentence spontaneously, and copy a complex polygon such as a Bender Gestalt figure. The maximum possible scores on the first and second parts are 21 and 9, respectively. A low MMSE score (≤24) may be related to cognitive decline.

The CDT assesses visuospatial, visuoconstructive, and memory abilities, as well as verbal and numerical knowledge [3]. A validated Spanish version of the CDT for the elderly population was used in this study [4]. The maximum possible score of the current version is 7 points. Lower scores indicate lower performance.

The DST is a Spanish version of the WAIS-III that measures attention and memory functions [5]. There are two versions: A) the digit span test forward version (DST-f), reflecting short-term memory, requires verbal repetition of a number sequence varying from 2 to 9 digits in the same order as provided by the interviewer; B) the DST-b, representing working memory, also requires verbal repetition of a number sequence from 2 to 8 digits, but in reverse order. For both tests, the task is finished when the participant fails two consecutive attempts. The score is proportional to the number of sequences completed. Higher scores indicate higher performance in memory function.

The Spanish Verbal Fluency Test (VFT) provides information about language and executive function [6]. Specifically, the animal category version of the semantic VFT (VFT-a) and the letter "p" version of the phonemic VFT (VFT-p) were used in this study. Participants are instructed to say as many words as possible related to the semantic category of animals for the VFT-a test and as many words as possible starting with the letter "p" for the VFT-p test. The total score was obtained from the list of properly stated words, with a time limit of 60 seconds for each test. The semantic VFT is influenced mainly by verbal abilities, whereas the phonemic VFT depends mainly on executive control [7].

The TMT consists of 25 circles spread over a sheet of paper and contains part A (TMT-A) and part B (TMT-B). In the TMT-A, participants were asked to connect consecutive numbers in ascending order (1, 2, 3, …) by drawing a line. In the TMT-B, they were asked to connect consecutive numbers and letters in an alternating numeric and alphabetic sequence (1–A, 2–B, 3–C, …). The TMT effectively discriminates cognitive dysfunction [8]. More specifically, the TMT-A assesses attention and processing speed [9] whereas the TMT-B requires more executive function, such as cognitive flexibility [10]. The score for each part is calculated according to the time taken to complete the task,

where more time spent indicates poorer performance. In this study, the TMT scores were referenced to normative data provided for the Spanish population [9].

References

1. Folstein MF, Folstein SE, McHugh PR (1975) “Mini-mental state.” J Psychiatr Res 12:189–198. https://doi.org/10.1016/0022-3956(75)90026-6
2. Blesa R, Pujol M, Aguilar M, et al (2001) Clinical validity of the ‘mini-mental state’ for Spanish speaking communities. Neuropsychologia 39:1150–1157. https://doi.org/10.1016/S0028-3932(01)00055-0
3. Paganini-Hill A, Clark LJ (2011) Longitudinal Assessment of Cognitive Function by Clock Drawing in Older Adults. Dement Geriatr Cogn Dis Extra 1:75–83. https://doi.org/10.1159/000326781
4. del Ser Quijan T, García De Yébenes MJ, Sánchez Sánchez F, et al (2004) [Cognitive assessment in the elderly. Normative data of a Spanish population sample older than 70 years]. Med Clin (Barc) 122:727–740. https://doi.org/10.1157/13062190
5. Rossi L, Neer C-R, Lopetegui S Escala de inteligencia para adultos de WECHSLER. WAIS-III Índice de comprensión verbal. Normas para los subtests: Vocabulario, analogías e información, para la Ciudad de La Plata Edades: 16 A 24 Años
6. Peña-Casanova J, Quiñones-Úbeda S, Quintana-Aparicio M, et al (2009) Spanish Multicenter Normative Studies (NEURONORMA Project): Norms for Verbal Span, Visuospatial Span, Letter and Number Sequencing, Trail Making Test, and Symbol Digit Modalities Test. Archives of Clinical Neuropsychology 24:321–341. https://doi.org/10.1093/ARCLIN/ACP038
7. Shao Z, Janse E, Visser K, Meyer AS (2014) What do verbal fluency tasks measure? Predictors of verbal fluency performance in older adults. Front Psychol 5:772. https://doi.org/10.3389/FPSYG.2014.00772/BIBTEX
8. Reitan RM (1955) The relation of the Trail Making Test to organic brain damage. J Consult Psychol 19:393–394. https://doi.org/10.1037/H0044509
9. Llinàs-Reglà J, Vilalta-Franch J, López-Pousa S, et al (2017) The Trail Making Test: Association With Other Neuropsychological Measures and Normative Values for Adults Aged 55 Years and Older From a Spanish-Speaking Population-Based Sample. Assessment 24:183–196. https://doi.org/10.1177/1073191115602552
10. Kortte KB, Horner MD, Windham WK (2010) The Trail Making Test, Part B: Cognitive Flexibility or Ability to Maintain Set? http://dx.doi.org/101207/S15324826AN0902_5 9:106–109. https://doi.org/10.1207/S15324826AN0902_5
