# Supplementary material for: Plasma levels of neurology-related proteins are associated with cognitive performance in an older population with overweight/obesity and metabolic syndrome
Source: GeroScience. 2023 Mar 25;45(4):2457–70. doi: 10.1007/s11357-023-00764-y (PMC10651568; doi:10.1007/s11357-023-00764-y)

Supplementary Figure S1 Scatterplot with means and SDs of the normalized expression levels of the 92 selected proteins in all participants

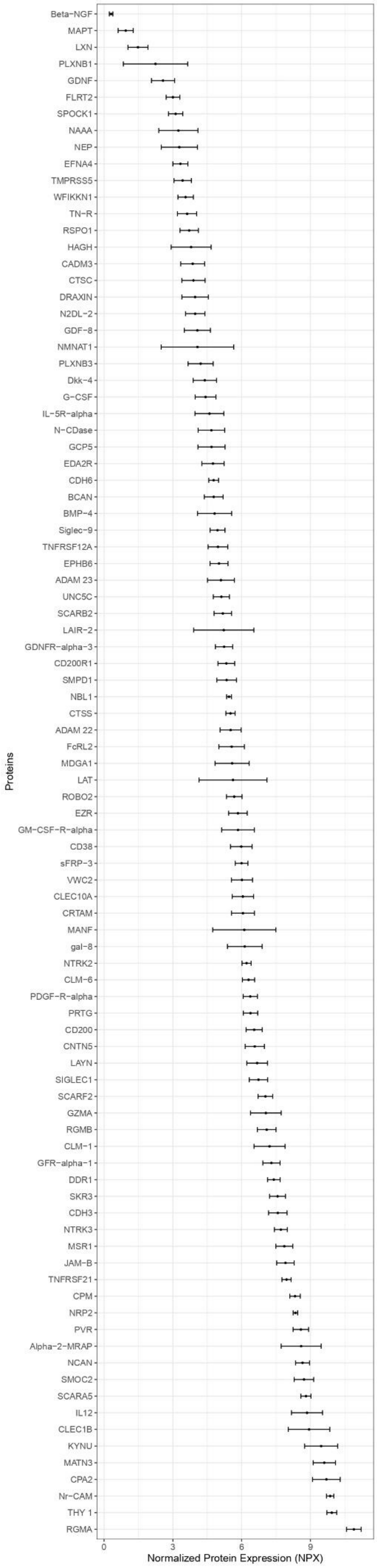

Supplement: Supplementary file 2 — ESM 2 [file 11357_2023_764_MOESM2_ESM.pdf]
