# Supplementary material for: Plasma levels of neurology-related proteins are associated with cognitive performance in an older population with overweight/obesity and metabolic syndrome
Source: GeroScience. 2023 Mar 25;45(4):2457–70. doi: 10.1007/s11357-023-00764-y (PMC10651568; doi:10.1007/s11357-023-00764-y)
Supplement: Supplementary file 3 — ESM 3 [file 11357_2023_764_MOESM3_ESM.pdf]

Supplementary Table S3 Pearson correlation coefficients between normalized protein expression (NPX) and cognitive z scores

Protein	zGCF	zgenCF	zExF	zAtt	zMMSE	zCDT	zVFTa	zVFTp	zTMTA	zTMTB	zDSTf	zDSTb
Siglec-9	**-0.23**	-0.17	-0.16	**-0.26**	**-0.19**	-0.09	-0.09	-0.17	**-0.24**	-0.15	-0.16	-0.09
NMNAT1	**-0.22**	**-0.20**	-0.16	**-0.21**	**-0.35**	0.00	-0.08	-0.13	-0.15	-0.06	-0.17	**-0.21**
HAGH	**-0.22**	**-0.33**	-0.07	**-0.21**	**-0.33**	**-0.21**	0.01	0.06	**-0.24**	-0.13	-0.07	-0.15
LXN[a]	**-0.22**	**-0.37**	-0.07	**-0.19**	**-0.36**	**-0.25**	0.04	0.01	-0.16	-0.08	-0.13	-0.17
gal-8	**-0.21**	-0.16	**-0.19**	-0.14	**-0.35**	0.07	-0.12	-0.16	-0.09	-0.10	-0.12	**-0.21**
Alpha-2-MRAP	**-0.20**	-0.08	**-0.20**	**-0.18**	-0.13	-0.01	-0.07	-0.14	**-0.20**	-0.15	-0.07	**-0.24**
IL12	**-0.20**	-0.17	-0.15	-0.17	-0.07	**-0.21**	-0.04	-0.15	-0.16	-0.16	-0.10	-0.11
PDGF-R-alpha	**-0.19**	-0.12	-0.11	**-0.26**	-0.10	-0.09	-0.06	-0.07	-0.27	-0.14	-0.12	-0.05
NAAA	**-0.19**	**-0.27**	-0.09	-0.16	**-0.33**	-0.11	0.01	-0.13	-0.16	-0.07	-0.08	-0.08
EDA2R	**-0.19**	-0.11	-0.15	**-0.22**	-0.06	-0.13	-0.07	-0.12	-0.18	**-0.20**	-0.15	-0.06
CLEC1B	**-0.18**	**-0.23**	-0.14	-0.06	**-0.38**	-0.01	-0.09	-0.12	-0.05	-0.07	-0.04	-0.15
LAT	**-0.18**	-0.16	-0.14	-0.14	**-0.36**	0.08	-0.10	-0.12	-0.11	-0.02	-0.12	**-0.19**
PLXNB3	-0.17	-0.17	-0.12	-0.13	**-0.36**	0.08	0.00	-0.11	-0.10	-0.07	-0.10	**-0.18**
MDGA1	-0.17	**-0.18**	-0.09	**-0.19**	-0.13	-0.15	-0.03	0.01	**-0.19**	-0.15	-0.10	-0.12
KYNU	-0.17	**-0.21**	-0.10	-0.14	**-0.27**	-0.08	-0.07	-0.13	**-0.21**	-0.08	0.00	-0.02
EZR	-0.16	**-0.22**	-0.06	**-0.18**	**-0.28**	-0.09	0.02	-0.08	**-0.18**	-0.01	-0.09	-0.10
SIGLEC1	-0.16	-0.12	-0.14	-0.14	-0.05	-0.15	-0.02	-0.11	-0.17	**-0.21**	-0.04	-0.07
GZMA	-0.16	**-0.20**	-0.05	**-0.19**	**-0.24**	-0.09	0.08	-0.11	-0.15	-0.07	-0.14	-0.05
CTSC	-0.16	**-0.26**	-0.04	-0.16	**-0.23**	**-0.20**	0.05	-0.07	-0.15	-0.04	-0.08	-0.07
CTSS	-0.15	**-0.23**	-0.02	**-0.18**	**-0.29**	-0.10	0.11	-0.01	-0.14	-0.09	-0.13	-0.08
Dkk-4	-0.15	-0.09	-0.10	**-0.20**	-0.09	-0.05	-0.03	-0.05	**-0.18**	-0.11	-0.14	-0.10
EFNA4	-0.14	-0.06	-0.10	**-0.19**	-0.04	-0.05	0.01	-0.15	**-0.22**	-0.14	-0.06	-0.03
MSR1	-0.14	-0.11	-0.10	-0.14	-0.07	-0.11	0.01	-0.09	-0.15	**-0.18**	-0.05	-0.03
THY 1	-0.14	-0.11	-0.10	-0.14	-0.03	-0.15	0.00	-0.08	-0.10	-0.15	-0.12	-0.07
TNFRSF21	-0.14	-0.08	-0.10	-0.17	-0.03	-0.10	0.02	-0.07	-0.16	-0.13	-0.09	-0.11
JAM-B	-0.13	-0.06	-0.10	-0.16	0.03	-0.12	0.01	-0.10	-0.15	-0.13	-0.09	-0.06
NTRK2	-0.12	-0.10	-0.05	**-0.18**	-0.15	-0.03	0.02	-0.03	-0.14	-0.04	-0.14	-0.09
MANF	-0.12	-0.07	-0.10	-0.12	**-0.26**	0.14	-0.11	-0.08	-0.08	0.06	-0.11	**-0.18**
UNC5C	-0.11	-0.10	-0.08	-0.11	-0.10	-0.06	-0.05	-0.05	-0.10	-0.10	-0.06	-0.04
GFR-alpha-1	-0.11	-0.01	-0.11	-0.13	-0.03	0.01	-0.03	**-0.20**	-0.11	-0.08	-0.09	-0.04
FLRT2	-0.11	-0.05	-0.06	**-0.19**	0.03	-0.10	-0.01	-0.05	**-0.22**	-0.10	-0.05	-0.02
GCP5	-0.11	**-0.18**	-0.06	-0.05	**-0.25**	-0.06	0.00	-0.04	-0.03	-0.03	-0.05	-0.11
LAIR-2	-0.11	0.00	-0.13	-0.13	-0.05	0.05	0.00	-0.14	-0.12	-0.13	-0.07	-0.11
SCARB2	-0.10	-0.06	-0.04	**-0.18**	0.02	-0.11	0.08	-0.05	-0.16	-0.14	-0.11	0.01
CPA2	-0.10	-0.15	-0.06	-0.06	-0.17	-0.08	-0.05	0.00	-0.09	-0.04	0.01	-0.07
GDNFR-alpha-3	-0.10	0.01	-0.07	**-0.20**	-0.02	0.03	0.01	-0.10	-0.14	-0.10	-0.17	-0.03
SKR3	-0.10	-0.01	-0.08	-0.17	0.04	-0.04	0.02	-0.11	**-0.21**	-0.14	-0.03	-0.03
N2DL-2	-0.10	-0.03	-0.10	-0.12	-0.05	-0.01	0.02	-0.14	-0.09	-0.12	-0.10	-0.05
CADM3	-0.09	-0.02	-0.07	-0.15	0.05	-0.08	0.02	-0.06	-0.08	-0.15	-0.16	-0.02
SCARA5	-0.09	-0.02	-0.07	-0.12	0.06	-0.09	-0.01	-0.12	-0.14	-0.07	-0.04	-0.01
TNFRSF12A	-0.09	0.01	-0.11	-0.09	0.01	0.01	-0.06	-0.13	-0.12	-0.14	-0.01	-0.02

FcRL2	-0.09	-0.05	-0.09	-0.07	0.01	-0.09	-0.07	-0.10	-0.09	-0.04	-0.02	-0.06
SCARF2	-0.08	0.01	-0.05	-0.16	0.04	-0.03	0.00	-0.07	-0.17	-0.09	-0.07	0.01
PVR	-0.08	-0.04	-0.12	0.01	-0.07	0.01	-0.09	-0.14	-0.01	-0.08	0.03	-0.05
GM-CSF-R-alpha	-0.07	-0.12	-0.02	-0.07	**-0.23**	0.04	-0.02	-0.02	0.00	0.02	-0.11	-0.03
Beta-NGF[a]	-0.07	-0.12	-0.04	-0.02	-0.16	-0.03	-0.09	0.00	0.00	0.00	-0.04	-0.04
DRAXIN	-0.07	-0.06	-0.02	-0.12	0.01	-0.10	0.05	-0.05	-0.13	-0.12	-0.05	0.07
LAYN	-0.06	0.06	-0.05	-0.15	0.11	-0.02	0.01	-0.06	-0.11	-0.10	-0.12	0.00
G-CSF	-0.06	0.07	-0.06	-0.15	0.05	0.06	-0.03	-0.13	-0.09	0.00	-0.15	-0.04
TN-R	-0.06	0.08	-0.08	-0.14	0.10	0.04	-0.05	-0.01	-0.06	-0.08	-0.16	-0.08
NCAN	-0.05	-0.02	0.00	-0.16	-0.01	-0.02	-0.01	0.07	-0.14	-0.04	-0.11	0.00
IL-5R-alpha	-0.05	-0.03	-0.04	-0.05	-0.02	-0.04	0.05	-0.04	-0.14	-0.08	0.07	-0.04
N-CDase	-0.05	-0.03	-0.01	-0.09	0.01	-0.07	0.00	0.01	-0.08	-0.07	-0.06	0.03
CD200R1	-0.05	-0.06	-0.02	-0.07	-0.08	-0.02	0.04	-0.09	-0.10	-0.01	0.01	0.00
VWC2	-0.04	0.00	-0.04	-0.07	0.04	-0.04	0.01	-0.13	-0.09	-0.04	-0.01	0.06
ADAM 22	-0.04	-0.02	0.03	-0.14	0.03	-0.06	0.08	-0.02	-0.14	0.02	-0.08	-0.01
RSPO1	-0.04	-0.02	0.02	-0.15	-0.02	-0.02	0.14	-0.01	-0.16	-0.08	-0.06	0.03
CD38	-0.03	0.02	0.01	-0.13	0.02	0.02	0.09	-0.06	-0.17	-0.04	-0.03	0.03
SMPD1	-0.03	0.02	0.01	-0.13	0.06	-0.03	0.07	-0.01	**-0.22**	-0.02	0.05	0.01
EPHB6	-0.03	-0.03	0.01	-0.09	0.06	-0.10	0.08	-0.03	-0.08	-0.04	-0.05	0.00
CD200	-0.03	-0.05	0.02	-0.08	-0.04	-0.04	0.04	0.04	-0.05	0.00	-0.08	0.00
MAPT[a]	-0.02	-0.07	-0.02	0.03	-0.06	-0.05	0.02	-0.12	0.05	0.02	-0.01	0.02
ROBO2	-0.02	0.08	0.02	-0.17	-0.01	0.14	0.11	-0.04	**-0.19**	-0.04	-0.07	0.04
PRTG	-0.01	0.02	0.00	-0.05	0.01	0.01	-0.01	0.01	-0.05	0.01	-0.04	-0.01
CLEC10A	-0.01	0.02	-0.01	-0.05	0.06	-0.03	0.03	-0.05	-0.07	-0.05	0.00	0.05
CLM-6	0.00	-0.03	0.02	-0.01	0.06	-0.10	0.14	-0.03	-0.05	-0.09	0.04	0.05
BCAN	0.00	0.05	0.03	-0.11	0.03	0.05	0.04	0.03	-0.09	0.03	-0.07	0.00
DDR1	0.00	0.04	0.02	-0.08	0.01	0.06	0.04	-0.02	-0.11	0.01	0.00	0.03
GDNF	0.01	0.06	-0.04	0.03	-0.05	0.14	-0.07	-0.04	-0.05	0.04	0.11	-0.05
sFRP-3	0.01	0.07	0.03	-0.09	0.06	0.06	0.04	0.07	-0.09	0.01	-0.05	-0.04
CLM-1	0.01	-0.05	0.05	-0.01	-0.06	-0.03	0.12	-0.08	-0.02	0.07	0.02	0.05
CDH6	0.02	-0.01	0.07	-0.03	0.07	-0.09	0.07	-0.01	-0.10	0.05	0.06	0.10
RGMB	0.03	0.04	0.05	-0.04	0.09	-0.03	0.10	0.00	-0.03	0.03	-0.03	0.01
TMPRSS5	0.03	0.12	0.04	-0.09	0.17	0.02	0.01	0.04	-0.08	-0.03	-0.06	0.10
CPM	0.03	-0.06	0.05	0.09	-0.11	0.00	0.04	0.01	0.03	0.13	0.13	-0.04
SPOCK1	0.03	0.08	0.08	-0.11	-0.01	0.13	0.03	0.06	**-0.26**	0.06	0.12	0.08
CDH3	0.05	0.11	0.03	-0.01	**0.18**	0.01	0.03	-0.11	-0.06	0.07	0.06	0.10
CRTAM	0.06	0.02	0.10	0.01	0.13	-0.09	0.13	0.04	0.01	0.04	0.00	0.09
CNTN5	0.06	0.06	0.06	0.01	0.12	-0.02	0.04	0.05	0.02	-0.01	-0.01	0.09
PLXNB1	0.06	0.00	0.07	0.05	0.04	-0.04	0.04	0.13	0.07	0.12	0.00	-0.08
NTRK3	0.06	0.06	0.09	-0.04	0.02	0.08	0.03	0.08	-0.08	0.08	0.02	0.08
Nr-CAM	0.07	0.09	0.10	-0.06	0.05	0.10	0.12	0.00	-0.08	0.16	-0.01	0.04
GDF-8	0.08	0.10	0.08	-0.01	0.09	0.08	0.07	-0.02	-0.04	0.13	0.03	0.05
NRP2	0.09	0.04	0.12	0.02	0.08	-0.01	0.10	0.09	0.00	0.15	0.03	0.04
NEP	0.09	0.10	0.10	0.00	0.01	0.16	0.09	-0.01	-0.08	0.11	0.11	0.11
RGMA	0.10	0.11	0.09	0.03	0.14	0.04	0.15	0.09	0.00	0.08	0.05	-0.05

WFIKKN1	0.10	0.04	0.14	0.04	0.08	-0.01	0.11	-0.01	-0.06	**0.20**	0.14	0.11
MATN3	0.11	0.10	0.12	0.01	0.09	0.07	0.10	0.12	-0.06	0.03	0.08	0.13
NBL1	0.14	0.04	0.13	0.17	-0.01	0.07	0.04	0.07	0.17	**0.23**	0.09	0.06
ADAM 23	0.14	**0.26**	0.08	0.04	**0.26**	0.16	0.11	-0.01	0.00	0.09	0.06	0.04
SMOC2	0.15	0.17	0.13	0.07	0.14	0.13	0.14	0.00	0.05	0.12	0.06	0.12
BMP-4	0.16	0.10	0.15	0.13	**0.18**	-0.01	0.07	**0.19**	0.10	**0.21**	0.11	0.00

zGCF, z score of global cognitive function; zgenGF, z score of general cognitive function domain; zExF, z score of executive function domain; zAtt, z score of attention domain; zMMSE, z score of Mini-Mental State Examination; zCDT, z score of Clock Drawing Test; zDST-f, z score of digit span test forward section; zDST-b, z score of digit span test backward section; zVFTa, z score of semantic Verbal Fluency Test animal category; zVFT-p, z score of phonemic Verbal Fluency Test letter “p” category; zTMT-A, z score of Trail Making Test part A; zTMT-B, z score of Trail Making Test part B.

Proteins are ordered by zGCF correlation coefficient.

Significant correlation coefficients (*p* value < 0.05) are indicated in bold.

[a] Proteins with a high percentage (>98%) of data values below the limit of detection (LOD).
