# Supplementary material for: Plasma levels of neurology-related proteins are associated with cognitive performance in an older population with overweight/obesity and metabolic syndrome
Source: GeroScience. 2023 Mar 25;45(4):2457–70. doi: 10.1007/s11357-023-00764-y (PMC10651568; doi:10.1007/s11357-023-00764-y)
Supplement: Supplementary file 4 — ESM 4 [file 11357_2023_764_MOESM4_ESM.pdf]

Supplementary Table S1 Distribution of participants with the lowest expression level (below the 10th percentile) for each protein

Protein name	lGCF	hGCF	p value*
IL12	2	11	0.013
NEP	10	3	0.052
MDGA1	3	10	0.052
PDGF-R-alpha	3	10	0.052
GDNF	4	9	0.166
Siglec-9	4	9	0.166
NBL1	9	4	0.166
NCAN	4	9	0.166
PRTG	4	9	0.166
PLXNB3	9	4	0.166
CD38	9	4	0.166
ADAM 22	9	4	0.166
ADAM 23	4	9	0.166
HAGH	4	9	0.166
LXN	9	4	0.166
GDF-8	4	9	0.166
TMPRSS5	4	9	0.166
CPM	9	4	0.166
CLEC10A	9	4	0.166
LAT	9	4	0.166
Nr-CAM	9	4	0.166
NRP2	8	5	0.405
UNC5C	8	5	0.405
SMOC2	8	5	0.405
SCARB2	5	8	0.405
CRTAM	5	8	0.405
SMPD1	8	5	0.405
sFRP-3	8	5	0.405
EPHB6	8	5	0.405
RGMB	8	5	0.405
SIGLEC1	5	8	0.405
MATN3	8	5	0.405
gal-8	5	8	0.405
WFIKKN1	8	5	0.405
CDH3	8	5	0.405
GFR-alpha-1	5	8	0.405
NTRK2	5	8	0.405
G-CSF	8	5	0.405
DRAXIN	5	8	0.405
BMP-4	8	5	0.405
CTSC	8	5	0.405
N-CDase	8	5	0.405
NAAA	8	5	0.405
TNFRSF21	5	8	0.405
CLM-1	8	5	0.405
SPOCK1	5	8	0.405
Dkk-4	5	8	0.405

NTRK3	8	5	0.405
LAIR-2	5	8	0.405
MANF	8	5	0.405
TN-R	5	8	0.405
NMNAT1	6	7	0.782
MAPT	7	6	0.782
CADM3	6	7	0.782
VWC2	7	6	0.782
CLM-6	7	6	0.782
EZR	7	6	0.782
EFNA4	6	7	0.782
ROBO2	6	7	0.782
RGMA	6	7	0.782
CPA2	6	7	0.782
MSR1	6	7	0.782
Alpha-2-MRAP	6	7	0.782
CNTN5	7	6	0.782
CLEC1B	7	6	0.782
RSPO1	7	6	0.782
BCAN	6	7	0.782
LAYN	7	6	0.782
THY 1	6	7	0.782
GM-CSF-R-alpha	6	7	0.782
Beta-NGF	6	7	0.782
SCARA5	6	7	0.782
CD200	6	7	0.782
GZMA	6	7	0.782
SCARF2	7	6	0.782
GDNFR-alpha-3	6	7	0.782
PVR	6	7	0.782
TNFRSF12A	7	6	0.782
SKR3	6	7	0.782
FLRT2	7	6	0.782
GCP5	7	6	0.782
FcRL2	6	7	0.782
IL-5R-alpha	6	7	0.782
CDH6	6	7	0.782
DDR1	7	6	0.782
JAM-B	7	6	0.782
CTSS	6	7	0.782
N2DL-2	6	7	0.782
PLXNB1	6	7	0.782
EDA2R	7	6	0.782
CD200R1	6	7	0.782
KYNU	6	7	0.782

*Each *p* value was calculated using the chi-square test.
