# Supplementary material for: Plasma levels of neurology-related proteins are associated with cognitive performance in an older population with overweight/obesity and metabolic syndrome
Source: GeroScience. 2023 Mar 25;45(4):2457–70. doi: 10.1007/s11357-023-00764-y (PMC10651568; doi:10.1007/s11357-023-00764-y)
Supplement: Supplementary file 5 — ESM 5 [file 11357_2023_764_MOESM5_ESM.pdf]

Supplementary Table S2 Descriptive analysis of normalized expression levels of the 92 selected proteins across all participants and within global cognitive function (GCF) categories

Protein	TOTAL	l-GCF	h-GCF	p value*
Alpha-2-MRAP	8.59 (8.44,8.75)	8.84 (8.58,9.11)	8.34 (8.20,8.47)	0.001
MDGA1	5.59 (5.46,5.72)	5.75 (5.57,5.92)	5.42 (5.24,5.61)	0.013
Siglec-9	4.95 (4.89,5.01)	5.02 (4.94,5.10)	4.88 (4.80,4.96)	0.014
HAGH	3.80 (3.64,3.95)	3.98 (3.74,4.22)	3.61 (3.43,3.79)	0.017
EDA2R	4.75 (4.67,4.84)	4.85 (4.71,4.98)	4.65 (4.55,4.75)	0.021
IL12	8.85 (8.73,8.97)	8.98 (8.83,9.13)	8.72 (8.54,8.90)	0.032
MSR1	7.86 (7.80,7.92)	7.93 (7.84,8.01)	7.79 (7.70,7.89)	0.043
SIGLEC1	6.74 (6.67,6.81)	6.80 (6.70,6.91)	6.67 (6.57,6.76)	0.053
PDGF-R-alpha	6.38 (6.32,6.43)	6.43 (6.35,6.51)	6.32 (6.25,6.40)	0.061
NBL1	5.45 (5.43,5.47)	5.43 (5.40,5.46)	5.47 (5.44,5.49)	0.074
TNFRSF21	7.96 (7.92,7.99)	7.99 (7.94,8.04)	7.93 (7.87,7.98)	0.081
FLRT2	3.00 (2.95,3.06)	3.05 (2.97,3.13)	2.96 (2.89,3.02)	0.082
DRAXIN	3.97 (3.87,4.07)	4.06 (3.92,4.20)	3.89 (3.74,4.03)	0.086
gal-8	6.14 (6.00,6.27)	6.25 (6.02,6.47)	6.02 (5.89,6.16)	0.096
GZMA	7.06 (6.94,7.17)	7.15 (6.95,7.35)	6.96 (6.84,7.08)	0.101
GFR-alpha-1	7.30 (7.23,7.36)	7.35 (7.25,7.45)	7.24 (7.16,7.33)	0.101
THY 1	9.93 (9.89,9.97)	9.96 (9.90,10.02)	9.90 (9.85,9.95)	0.105
CD200R1	5.33 (5.27,5.40)	5.38 (5.30,5.47)	5.28 (5.19,5.37)	0.107
KYNU	9.47 (9.34,9.59)	9.57 (9.37,9.76)	9.36 (9.21,9.52)	0.108
EFNA4	3.33 (3.27,3.39)	3.38 (3.29,3.46)	3.28 (3.20,3.36)	0.109
NEP	3.28 (3.14,3.42)	3.17 (2.97,3.37)	3.39 (3.20,3.58)	0.114
NAAA	3.24 (3.09,3.39)	3.36 (3.10,3.62)	3.13 (2.98,3.27)	0.124
N2DL-2	3.97 (3.90,4.05)	4.03 (3.92,4.14)	3.92 (3.82,4.02)	0.126
PVR	8.58 (8.52,8.64)	8.63 (8.55,8.71)	8.54 (8.45,8.62)	0.133
SCARA5	8.80 (8.77,8.84)	8.83 (8.78,8.89)	8.78 (8.72,8.83)	0.134
TMPRSS5	3.43 (3.36,3.49)	3.48 (3.39,3.56)	3.38 (3.27,3.48)	0.137
RSPO1	3.71 (3.64,3.78)	3.76 (3.66,3.87)	3.66 (3.57,3.75)	0.157
SCARF2	7.04 (6.98,7.09)	7.08 (6.99,7.16)	7.00 (6.93,7.07)	0.172
GCP5	4.69 (4.58,4.79)	4.76 (4.59,4.92)	4.61 (4.49,4.74)	0.172
PLXNB3	4.21 (4.12,4.31)	4.28 (4.11,4.45)	4.15 (4.06,4.23)	0.179
TNFRSF12A	4.97 (4.89,5.04)	5.02 (4.91,5.13)	4.92 (4.81,5.02)	0.181
GDNFR-alpha-3	5.23 (5.17,5.30)	5.28 (5.18,5.37)	5.19 (5.10,5.28)	0.186
NTRK2	6.21 (6.18,6.25)	6.24 (6.19,6.28)	6.19 (6.14,6.24)	0.191
NRP2	8.34 (8.33,8.36)	8.33 (8.31,8.36)	8.35 (8.33,8.38)	0.195
JAM-B	7.91 (7.84,7.98)	7.95 (7.85,8.05)	7.87 (7.78,7.96)	0.199

CRTAM	6.06 (5.97,6.15)	6.11 (5.99,6.24)	6.00 (5.88,6.12)	0.199
LXN[a]	1.48 (1.40,1.56)	1.53 (1.38,1.68)	1.43 (1.39,1.47)	0.201
CLEC1B	8.94 (8.78,9.10)	9.04 (8.78,9.30)	8.84 (8.66,9.02)	0.215
EZR	5.84 (5.77,5.91)	5.88 (5.76,6.00)	5.79 (5.71,5.88)	0.223
TN-R	3.62 (3.55,3.69)	3.67 (3.56,3.77)	3.58 (3.47,3.68)	0.235
SCARB2	5.18 (5.11,5.25)	5.22 (5.12,5.31)	5.14 (5.04,5.23)	0.241
NMNAT1	4.07 (3.80,4.35)	4.24 (3.78,4.69)	3.91 (3.59,4.23)	0.242
FcRL2	5.57 (5.47,5.66)	5.62 (5.47,5.76)	5.51 (5.38,5.64)	0.276
SKR3	7.57 (7.51,7.63)	7.60 (7.51,7.69)	7.53 (7.45,7.62)	0.277
LAT	5.62 (5.36,5.88)	5.76 (5.34,6.18)	5.48 (5.17,5.80)	0.292
Dkk-4	4.39 (4.31,4.48)	4.44 (4.33,4.56)	4.35 (4.21,4.49)	0.297
PLXNB1	2.24 (2.00,2.49)	2.12 (1.88,2.35)	2.37 (1.94,2.81)	0.305
CPA2	9.69 (9.59,9.80)	9.75 (9.59,9.90)	9.64 (9.50,9.78)	0.309
LAIR-2	5.22 (4.99,5.45)	5.33 (5.03,5.64)	5.10 (4.76,5.45)	0.323
DDR1	7.40 (7.35,7.45)	7.42 (7.36,7.49)	7.38 (7.31,7.45)	0.356
CTSS	5.51 (5.48,5.55)	5.53 (5.47,5.59)	5.50 (5.46,5.54)	0.356
GDNF	2.57 (2.48,2.66)	2.61 (2.49,2.73)	2.53 (2.40,2.66)	0.382
BMP-4	4.82 (4.69,4.95)	4.77 (4.58,4.95)	4.88 (4.69,5.07)	0.384
GM-CSF-R-alpha	5.84 (5.72,5.97)	5.90 (5.73,6.06)	5.79 (5.60,5.97)	0.389
CD38	5.98 (5.90,6.07)	6.02 (5.91,6.13)	5.95 (5.83,6.07)	0.395
SMOC2	8.72 (8.64,8.79)	8.69 (8.58,8.80)	8.75 (8.65,8.85)	0.422
CNTN5	6.57 (6.50,6.64)	6.54 (6.44,6.64)	6.60 (6.49,6.71)	0.431
IL-5R-alpha	4.60 (4.49,4.71)	4.64 (4.49,4.78)	4.55 (4.39,4.72)	0.451
CTSC	3.90 (3.81,3.99)	3.93 (3.78,4.08)	3.87 (3.77,3.97)	0.462
Nr-CAM	9.86 (9.83,9.89)	9.87 (9.83,9.91)	9.85 (9.81,9.89)	0.483
CADM3	3.87 (3.77,3.96)	3.90 (3.77,4.03)	3.83 (3.70,3.96)	0.484
N-CDase	4.68 (4.58,4.78)	4.72 (4.58,4.85)	4.64 (4.49,4.80)	0.485
LAYN	6.67 (6.60,6.75)	6.70 (6.59,6.82)	6.65 (6.54,6.75)	0.485
PRTG	6.39 (6.33,6.44)	6.40 (6.33,6.48)	6.37 (6.29,6.45)	0.497
NCAN	8.66 (8.60,8.71)	8.67 (8.61,8.74)	8.64 (8.55,8.72)	0.507
SPOCK1	3.12 (3.07,3.18)	3.14 (3.06,3.22)	3.10 (3.03,3.18)	0.515
GDF-8	4.07 (3.97,4.17)	4.10 (3.96,4.24)	4.04 (3.89,4.18)	0.527
SMPD1	5.35 (5.27,5.42)	5.37 (5.25,5.49)	5.32 (5.22,5.42)	0.542
EPHB6	5.01 (4.95,5.08)	5.03 (4.93,5.13)	4.99 (4.90,5.09)	0.562
CLM-1	7.22 (7.10,7.33)	7.18 (6.99,7.38)	7.25 (7.12,7.38)	0.572
CDH3	7.58 (7.51,7.65)	7.60 (7.49,7.70)	7.56 (7.46,7.65)	0.576
ADAM 22	5.52 (5.44,5.60)	5.54 (5.42,5.66)	5.50 (5.38,5.61)	0.588
UNC5C	5.12 (5.05,5.18)	5.13 (5.04,5.22)	5.10 (5.01,5.18)	0.598
ROBO2	5.68 (5.62,5.74)	5.69 (5.60,5.78)	5.66 (5.58,5.74)	0.598

G-CSF	4.42 (4.35,4.50)	4.44 (4.33,4.56)	4.40 (4.29,4.52)	0.631
VWC2	6.01 (5.93,6.09)	6.03 (5.91,6.15)	5.99 (5.88,6.11)	0.640
WFIKKN1	3.56 (3.50,3.62)	3.57 (3.48,3.66)	3.54 (3.46,3.62)	0.646
CPM	8.33 (8.29,8.37)	8.32 (8.25,8.39)	8.34 (8.30,8.38)	0.656
MATN3	9.60 (9.52,9.69)	9.58 (9.47,9.70)	9.62 (9.49,9.75)	0.659
ADAM 23	5.10 (4.99,5.20)	5.12 (4.95,5.28)	5.07 (4.95,5.19)	0.677
MANF	6.11 (5.87,6.35)	6.16 (5.80,6.52)	6.07 (5.74,6.40)	0.703
CLM-6	6.30 (6.26,6.35)	6.31 (6.24,6.38)	6.29 (6.23,6.36)	0.738
BCAN	4.78 (4.71,4.85)	4.79 (4.70,4.89)	4.77 (4.66,4.88)	0.762
RGMA	10.89 (10.84,10.95)	10.89 (10.80,10.97)	10.90 (10.83,10.98)	0.770
sFRP-3	5.99 (5.95,6.04)	6.00 (5.93,6.07)	5.99 (5.92,6.05)	0.784
MAPT[a]	0.94 (0.88,1.00)	0.93 (0.87,1.00)	0.95 (0.85,1.05)	0.798
NTRK3	7.71 (7.66,7.76)	7.71 (7.64,7.78)	7.72 (7.65,7.79)	0.843
RGMB	7.09 (7.02,7.16)	7.10 (6.99,7.20)	7.08 (6.99,7.18)	0.860
CLEC10A	6.05 (5.97,6.13)	6.06 (5.93,6.19)	6.05 (5.95,6.15)	0.876
CD200	6.54 (6.48,6.61)	6.55 (6.46,6.63)	6.54 (6.45,6.63)	0.936
CDH6	4.78 (4.74,4.82)	4.78 (4.73,4.83)	4.78 (4.73,4.83)	0.983
Beta-NGF[a]	0.31 (0.29,0.32)	0.31 (0.29,0.33)	0.31 (0.29,0.33)	0.985

Descriptive data are expressed as the mean (confidence interval).

* Each *p* value was calculated using Student's t test.

[a] Proteins with a high percentage (>98%) of data values below the limit of detection (LOD).
